# COMMENTARY ON “EFFECT OF BASELINE VALUES ON INPATIENT REHABILITATION OUTCOMES AFTER TOTAL KNEE ARTHROPLASTY: A RETROSPECTIVE OBSERVATIONAL STUDY”

**DOI:** 10.2340/jrm.v57.43092

**Published:** 2025-04-08

**Authors:** Ahmad Furqan ANJUM

**Affiliations:** Department of Orthopaedics, Shaikh Zayed Postgraduate Medical Institute (SZPGMI), Lahore, Pakistan

*To the Editor*,

I would like to commend the authors, Missmann & Fischer, for their valuable contribution in evaluating inpatient rehabilitation outcomes following total knee arthroplasty (TKA) in their retrospective observational study (1). The study provides insightful findings on the rehabilitation progress of patients with different baseline values and highlights the significance of inpatient rehabilitation programmes. The use of patient-reported outcome measures (PROMs) and physical performance-based outcomes (PPOs) adds credibility to their analysis. Their attempt to address a clinically relevant topic is noteworthy and beneficial for optimizing post-TKA rehabilitation strategies. However, while the study offers important insights, several methodological flaws and limitations, beyond those already mentioned by the authors, warrant discussion.

First, the study assumes that a standardized rehabilitation protocol is equally effective across all participants. However, evidence suggests that individualized rehabilitation programmes tailored to patients’ comorbidities and functional baselines yield better outcomes (2). By ignoring this variability, the study risks overgeneralizing its conclusions. Thus, future studies should compare standardized vs individualized rehabilitation protocols. For instance, stratifying patients based on frailty indices or comorbidities (3) may yield actionable insights. Second, while the study employs PROMs (e.g., WOMAC, EQ-5D-5L) and PPOs (e.g., ROM, TUG), it could benefit from incorporating more objective functional assessments, such as gait analysis or muscle strength tests. Stratford et al. (4) argue that PROMs often fail to capture nuanced functional improvements. Thus, the study can complement PROMs and PPOs with biomechanical or imaging-based assessments to provide a holistic evaluation. Future retrospective studies could integrate gait analysis through video-based motion tracking, analysing pre-existing rehabilitation footage or standardized assessments at follow-up visits. Wearable inertial sensors, as demonstrated by Mizner et al. (5), provides objective gait parameters such as stride length and cadence, offering deeper insight into functional improvements post-TKA. Motion capture technology or mobile-based gait analysis applications could also be utilized to assess dynamic balance and functional mobility retrospectively. Incorporating biomechanical assessments alongside patient-reported outcomes would allow for a more comprehensive evaluation of recovery trajectories. Third, the study focuses primarily on physical and functional recovery, but it does not account for psychosocial factors such as patient motivation, mental health, and social support, which significantly influence rehabilitation outcomes. Research by Haraldstad et al. and Choi & Ra (6, 7) suggests that anxiety, depression, and self-efficacy play a crucial role in post-TKA rehabilitation. Thus, future studies should integrate psychological assessments, such as the Hospital Anxiety and Depression Scale (HADS), to assess the impact of mental health on rehabilitation outcomes. Fourth, the study does not account for the degree of functional decline before surgery, which can significantly influence postoperative rehabilitation outcomes. Patients with a more severe decline before surgery may exhibit greater improvements, creating an illusion of rehabilitation effectiveness. Mizner et al. (5) demonstrated that preoperative strength and function strongly predict postoperative mobility. Thus, future studies should include preoperative functional assessments to establish a clearer baseline for comparison. Fifth, the study evaluates outcomes only during inpatient rehabilitation and does not account for whether patients continued structured rehabilitation post-discharge. However, long-term functional outcomes following TKA are significantly influenced by adherence to structured rehabilitation programmes after hospital discharge. Several studies, including Chaudhry et al. (2), indicate that ongoing supervised rehabilitation or home exercise programmes significantly improve mobility, strength, and patient satisfaction compared with those who do not continue structured rehabilitation. For instance, Buhagiar et al. (8) conducted a randomized controlled trial (RCT) comparing inpatient rehabilitation with a home-based programme and found that patients who adhered to structured rehabilitation, whether inpatient or home-based, demonstrated superior functional improvements at 6 months post-surgery.

In conclusion, while Missmann and Fischer’s study (1) provides valuable insights into inpatient rehabilitation following TKA, several methodological limitations reduce the generalizability and applicability of their findings. Addressing the issues of protocol homogeneity, reliance on PROMs, preoperative functional decline, psychosocial influences, and post-discharge rehabilitation adherence would improve the robustness of future research. Additionally, incorporating individualized rehabilitation strategies, objective biomechanical assessments, and surgical variables would allow for a more comprehensive understanding of patient recovery. A multicentre, prospective design with long-term follow-up would also enhance the validity of rehabilitation recommendations. By refining these aspects, future studies can more effectively determine the optimal rehabilitation strategies tailored to individual patient needs, ultimately improving clinical outcomes and post-TKA quality of life.
