# Supplementary material for: COMMENTARY ON “EFFECT OF BASELINE VALUES ON INPATIENT REHABILITATION OUTCOMES AFTER TOTAL KNEE ARTHROPLASTY: A RETROSPECTIVE OBSERVATIONAL STUDY”
Source: J Rehabil Med. 2025 Apr 8;57:43092. doi: 10.2340/jrm.v57.43092 (PMC11995727; doi:10.2340/jrm.v57.43092)
Supplement: Related texts

# REPLY TO THE COMMENTARY ON “EFFECT OF BASELINE VALUES ON INPATIENT REHABILITATION OUTCOMES AFTER TOTAL KNEE ARTHROPLASTY: A RETROSPECTIVE OBSERVATIONAL STUDY”

**Authors:** Martin MISSMANN, Michael J. FISCHER

**Affiliations:** Austrian Workers’ Compensation Board, Innsbruck, and Ludwig Boltzmann Institute for Rehabilitation Research, Vienna, Austria

*To the Editor,*

We appreciate the insightful commentary and the valuable suggestions provided regarding our study on inpatient rehabilitation outcomes following total knee arthroplasty (TKA). We acknowledge the importance of assessing whether all patients should receive the same intensity and duration of rehabilitation. How-ever, in Austria, the framework for rehabilitation is standardized, limiting the degree of individualized treatment to adjustments in the amount of individual or physical therapy.

Despite these standardized conditions, our study demonstrates that patients show varying outcomes. This homogeneity in the rehabilitation environment actually strengthens the study design by reducing variability. Additionally, patients with higher frailty indices or complex comorbidities, who require more intensive care, were not included, further contributing to a relatively homogeneous sample.

We recognize that advanced gait analysis techniques, such as video-supported assessments or electronic measuring systems, could provide more precise functional data. However, implementing such technologies in large patient groups within rehabilitation settings poses significant logistical challenges. Therefore, we relied on established functional measures like the 6MWT and TUG, which reliably indicate mobility and functionality.

Regarding the impact of psychological status on functional outcomes, we concur that this is a relevant factor. Depressive symptoms can indeed influence pain perception and functional assessments. Integrating tools like the HADS would enhance the evaluation of psychological influences on rehabilitation outcomes. Where feasible, future programmes should consider including such assessments.

Our study focused on outcomes during the inpatient rehabilitation period. We acknowledge that functional improvements, particularly in joint mobility, may continue to evolve months after rehabilitation. This dynamic progression is noted in our study and will be explored further in future research with different study designs.

One key finding of our study was that a substantial proportion of patients may not require inpatient rehabilitation after TKA. This highlights the need for more precise preoperative and postoperative assessments to optimize rehabilitation allocation. At the same time, this approach is crucial to ensure that patients with significant impairments receive the necessary rehabilitation services, especially in the context of increasing healthcare cost pressures.

We appreciate the opportunity to address these points and thank the reviewer for their thoughtful feedback, which contributes to the ongoing dialogue aimed at optimizing post-TKA rehabilitation strategies.
